# CRISPR/Cas9 revitalizes adoptive T-cell therapy for cancer immunotherapy

**DOI:** 10.1186/s13046-021-02076-5

**Published:** 2021-08-26

**Authors:** Sasan Ghaffari, Nastaran Khalili, Nima Rezaei

**Affiliations:** 1 Cancer Immunology Project (CIP), Universal Scientific Education and Research Network (USERN), Tehran, Iran; 2 Department of Hematology, School of Allied Medical Sciences, Tehran University of Medical Sciences, Tehran, Iran; 3 Research Center for Immunodeficiencies, Children’s Medical Center, Tehran University of Medical Sciences, Dr. Qarib St, Keshavarz Blvd, Tehran, 14194 Iran; 4 Department of Immunology, School of Medicine, Tehran University of Medical Sciences, Tehran, Iran; 5 Network of Immunity in Infection, Malignancy and Autoimmunity (NIIMA), Universal Scientific Education and Research Network (USERN), Stockholm, Sweden

**Keywords:** Adoptive immunotherapy, Cancer, Cas enzymes, Clustered regularly interspaced short palindromic repeats, CRISPR/Cas-9, T-lymphocytes, Gene editing

## Abstract

Cancer immunotherapy has gained attention as the supreme therapeutic modality for the treatment of various malignancies. Adoptive T-cell therapy (ACT) is one of the most distinctive modalities of this therapeutic approach, which seeks to harness the potential of combating cancer cells by using autologous or allogenic tumor-specific T-cells. However, a plethora of circumstances must be optimized to produce functional, durable, and efficient T-cells. Recently, the potential of ACT has been further realized by the introduction of novel gene-editing platforms such as the CRISPR/Cas9 system; this technique has been utilized to create T-cells furnished with recombinant T-cell receptor (TCR) or chimeric antigen receptor (CAR) that have precise tumor antigen recognition, minimal side effects and treatment-related toxicities, robust proliferation and cytotoxicity, and nominal exhaustion. Here, we aim to review and categorize the recent breakthroughs of genetically modified TCR/CAR T-cells through CRISPR/Cas9 technology and address the pearls and pitfalls of each method. In addition, we investigate the latest ongoing clinical trials that are applying CRISPR-associated TCR/CAR T-cells for the treatment of cancers.

## Background

Cancer is the second leading cause of death worldwide. According to the American Cancer Society (ACS), in 2021, roughly 600,000 cancer-related deaths and 1.9 million incidental cases are projected to occur in the US alone [1]. Traditional cancer treatment methods such as surgery, radiotherapy, and/or chemotherapy have come a long way in hindering cancer progression, enabling tumor ablation and causing remissions in various cancers; however, a tendency for relapse or drug resistance often leads to a poor prognosis. Chemotherapy is essentially toxic to not only cancer cells but also to normal bystander cells. Its lack of specificity could lead to detrimental side effects, and in some cases, it can even contribute to the death of patients. Since radiotherapy is also associated with the same pitfall as chemotherapy, the need for detecting novel precision cancer therapies is necessitated [2]. Immunotherapy, in which the immune system is manipulated to fight cancer more efficiently, is a novel treatment modality that has gained extensive attention over the past years. Adoptive cell therapy (ACT) is an immunotherapeutic approach, which seeks to harness immune cells, particularly T-cells, for fighting tumor cells. Tumor-infiltrating lymphocyte (TIL) therapy was one of the first types of ACT that aimed at isolating infiltrated lymphocytes from tumors, culturing and expanding them in vitro, and then reinfusing them back to the patient. The difficulties associated with the isolation and expansion of TILs, and the modest anti-tumor effects of this modality led to the generation of engineered T-cell receptor (TCR)-T cells. However, the reliance of TCR-T cells on major histocompatibility molecules (MHCs) to recognize tumor antigens was a major flaw of this method as tumor cells were capable of downregulating MHC expression to evade immune surveillance [3]. Later on, the introduction of synthetic chimeric antigen receptor (CAR) T-cells transformed the field of immunotherapy once again, allowing immune cells to recognize specific tumor antigens in the absence of MHCs. This approach yielded positive results with up to 90% complete remission (CR) being reported in patients with acute B-cell lymphoblastic leukemia (B-ALL) using anti-CD19 CAR T-cell therapy [4]. Still, adverse side effects, such as neurotoxicity, T-cell exhaustion, and low-quality cells obstructed ACT from reaching its full potential [5]. The generation of efficient CAR T-cells and TCR T-cells is bound to the successful engineering of T-cells that are inherently less prone to immune evasion and suppression by cancer cells and cause less off-target toxicity; genetic modifications can pave the way for generating such cells.

Genome editing is a branch of genetic engineering by which DNA sequences are altered through insertions, deletions, or other modifications in the genome of the cell. This technique can be used as a tool to insert a desired genomic sequence or delete an impaired gene. After the advent of genome editing in the 1990s and following its recent developments, this method has gained attention for precisely targeting cancer-related genes and subsequently, quelling tumor growth. The long-term effects of genome editing can potentially unshackle patients from multiple sessions of therapy and its precision can avoid the unfavorable off-target cytotoxic side effects that came with previous treatments [6].

Over the years, different methods have been devised to achieve and improve genetic engineering. Zinc finger nucleases (ZFNs) and transcription activator-like effector nucleases (TALENs) are two prominent conventional genome editing platforms used for creating various cancer models. Both methods utilize a zinc finger protein or a transcription activator-like effector (TALE) domain to recognize and bind to DNA at a specific sequence and an endonuclease to cut the DNA, creating a DNA double-stranded break (DSB) [7]. The cellular DNA repair system then proceeds to fix the DNA at the DSB using a template. Simultaneously, the template is artificially introduced in the cell using a vector that contains the desired genetic sequence. This process culminates in replacing a DNA sequence with the sequence of choice. However, producing endonucleases that are specific for the target sequence is cumbersome and time-consuming [8]. Although there have been a few clinical trials using ZFN as a therapeutic approach (mostly in HIV treatment), further application of ZFN has been hindered, mainly due to its off-target mutation potential [9]. Due to a longer DNA recognition site, TALENs are less prone to off-target mutations, less time-consuming, and more specific as opposed to ZFNs. In addition, TALEs are easier to construct. Nevertheless, the larger and repetitive structure of TALENs makes their delivery to cells using lentiviruses or single adeno-associated virus (AAV) particles more difficult [10].

Lately, another installment of genome editing has been introduced that revolves around clustered regularly interspaced short palindromic repeats (CRISPR) and its associated protein 9 (Cas9); this is a more feasible technique than the two previous methods. The novelty of the CRISPR/Cas9 system is that it utilizes a single-guide RNA (gRNA) to pinpoint the location of the target DNA sequence and then instructs the Cas9 protein to cut the DNA and create a DSB. Because there is no need for creating a sequence-specific endonuclease, CRISPR/Cas9 can circumvent the arduous process of its predecessors and become a versatile platform for modeling various diseases including cancer [6]. CRISPR/Cas9 may even be utilized for the treatment of cancers caused by epigenetic alterations [11].

In this review, we investigate from an immunotherapy point of view, the current knowledge and strategies regarding the application of CRISPR/Cas9 for adoptive cell therapy of cancer. We also discuss in detail the challenges associated with the transition of this technique from bench to bedside and its further development. Although we have not discussed the implications of CRISPR/Cas9 in NK cell therapy [12], we have exhaustively reviewed the current in vivo/in vitro studies and clinical trials of ACT that have taken advantage of CRISPR/Cas9 platform to boost the efficacy of engineered T-cells.

## The CRISPR/Cas9 system (origin, mechanism, advantages)

**Fig. 1:**
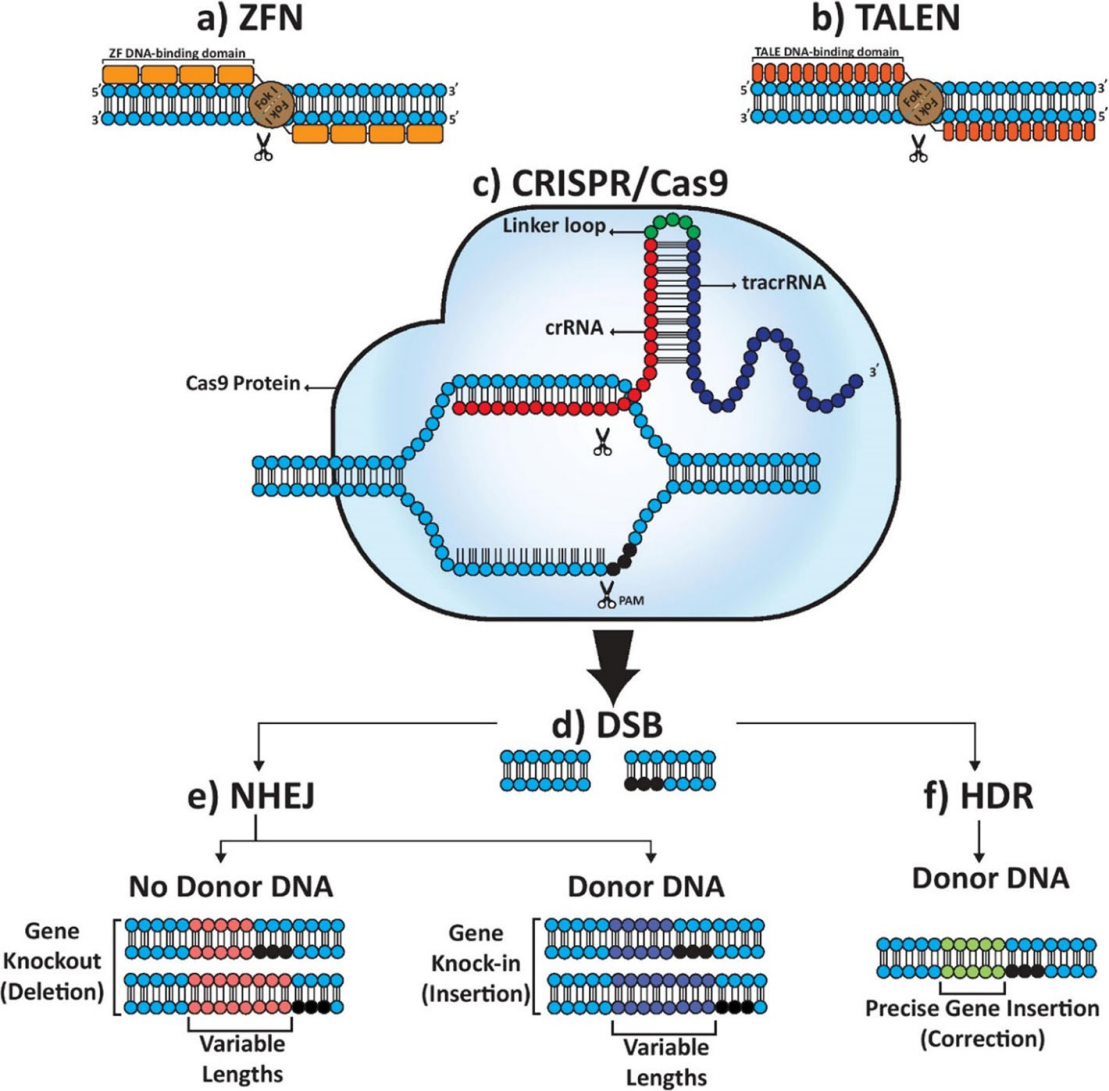
The most prominent genome editing platforms for DSB induction and its subsequent repair. ZFNs (**a**) and TALENs (**b**) utilize their respective DNA-binding domain to distinguish target sequences and form a dimer. Dimerized FokI proceeds to cleave the sequence. Cas9-related DSB generation requires a PAM sequence (**c**). NHEJ and/or HDR can both participate in repairing the DSBs (**d**). In the absence of donor DNA, NHEJ repair results in gene knockout. When donor DNA is available, it is cleaved by the nucleases at the same time, producing overhangs consistent with the cleaved target sequence. NHEJ uses this template to fill the gap and produce knock-ins (**e**). Similarly, HDR can repair DSBs to insert or correct genes when donor DNA is present but with more precise substitution (**f**)

The most recent addition to gene editing tools has been the CRISPR/Cas9 system, which is essentially a bacterial defensive mechanism against foreign DNA. CRISPR was initially discovered in the late 1980s as an unusual genetic structure composed of alternating repetitive and non-repetitive DNA sequences; however, the function of this complex system remained unknown until 2005 [13]. Genomic analyses revealed that the CRISPR and Cas proteins act as an acquired immune system and protect the prokaryotic DNA against phage and plasmid DNA through an RNA-guided DNA cleavage system [14]. When a foreign DNA invades a bacterium, a segment of its DNA incorporates in the CRISPR locus as a spacer. The locus is then transcribed to CRISPR pre-RNA (crRNA) which attaches to constitutively-formed *trans*-activating RNA (tracrRNA) to be modified into gRNA by CRISPR-associated proteins. When gRNA binds to the REC I domain of an inactive Cas9 complex, the complex becomes activated, resulting in gRNA and its complementary single-stranded DNA to form a heteroduplex. The HNH and RuvC nuclease domains of Cas9 subsequently proceed to cleave the complementary and non-complementary DNA strand, respectively [15]. A REC II domain also exists, the function of which is still unknown. This Cas protein which is comprised of multiple subunits to degrade DNA is categorized as class 1; the class 2 CRISPR-Cas system utilizes one single large Cas protein for the same purpose. Class 1 is divided into type I, III, and IV, and class 2 is divided into type II, V, and VI. Each type has its signature Cas protein. Cas9 belongs to type II of class 1 CRISPR-Cas system and is derived from *S. pyogenes* (SpCas9) [16]. Cas9 coupling to target sequence requires a protospacer adjacent motif (PAM), a small 3–8 bp DNA sequence in the invading genome, absent in the bacterial host genome. Thus, PAM is pivotal in distinguishing bacterial self and non-self DNA and successful Cas9 binding. This could be a major hurdle in gene editing settings since a PAM sequence downstream of the gRNA target sequence is necessary. The arginine-rich PAM-interacting domain of Cas9 confers PAM specificity. The first 20 bp of gRNA are guide sequences crucial in target DNA recognition [17]. This section itself is made up of non-seed and seed sequence, the latter of which is considered the most important sequence of gRNA (Fig. 1).

Unlike ZFN and TALEN, which are dependent on elaborate protein production for each specific gene sequence, the CRISPR/Cas9 system relies on gRNAs, making it a more flexible platform. The Cas9 protein remains constant, while the gRNA is conveniently tailored to each gene. Another advantage of the CRISPR/Cas9 system is that it makes simultaneous gene editing at multiple loci possible, allowing for a more efficient and scalable platform compared to its predecessors [18]. The Cas9 and its correspondent gRNA can be delivered into cells on the same plasmids or different ones. Table 1 compares in detail the most commonly used genome editing systems at the present time.

**Table 1:** Comparison of genome editing techniques (ZFN vs TALEN vs CRISPR)

	ZFN	TALEN	CRISPR
**Recognition Site**	Zinc finger proteins	RVD regions of TALE protein	Single guide RNA
**Target Sequence Length (bp†)**	9–12	14–20	~ 20
**Nuclease**	FokI nuclease	FokI nuclease	Cas9 protein
**Engineering Feasibility**	Low	Higher	Highest
**Cytotoxicity**	High	Moderate	Moderate
**Susceptibility to Immune Reactions**	Low	Low	Moderate
**Clinical Trials Usage**	Moderate	Low	High
**Enzyme Engineering**	Difficult	Easy	Very easy
**Means of Recognition**	Protein	Protein	RNA-DNA pairing and then protein
**Efficiency**	Moderate	Moderate	High
**Specificity**	Low	Moderate	High
**Multiple Gene Editing**	No	Yes	Yes
**Gene Editing in Babies**	No	No	Yes
**RNA Editing**	No	No	Yes
**Vector Delivery**	Easy due to small size	Difficult due to large size	Moderate as SpCas9 is relatively large
**Cost**	Low	High	Low

*bp* Base pair, *ZFN* Zinc-finger nuclease, *TALEN* Transcription activator-like effector nuclease, *CRISPR* Clustered regularly interspaced short palindromic repeat

## Gene editing in human T-cells

Adoptive T-cell therapy, which extracts, modifies, and then reinfuses the modified immune cells back into the patient’s body, is an effective and promising tool in cancer treatment. This method came to attention after two of its products, axicabtagene ciloleucel or Axi-cel (yescarta, Kite) and Tisagenlecleucel (kymriah, Novartis), were approved by the FDA for the treatment of diffuse large B-cell lymphoma (DLBCL) and CD19^+^ B-ALL [19]. These agents were modified using a chimeric antigen receptors (CAR) construct; in this method, T-cells are redirected toward tumor cells by introducing a modified T-cell receptor (TCR), which overcomes the reliance on isolating and expanding pre-existing tumor-infiltrating lymphocytes. Each CAR T-cell or TCR T-cell therapy has its own merits and drawbacks; for instance, CARs can only recognize cell surface antigens but without the need for MHC presentation; however, modified TCRs recognize both cell surface- and intracellular-derived peptides but do so in an MHC-dependent manner.

In earlier studies, Okamoto et al. used small interfering RNA (siRNA) to knockdown endogenous TCR expression, enhancing the lysis of tumor cells [20]. ZFNs were the first gene-editing method to silence endogenous TCRs and create superior antigen-specific T-cells [21]. On different occasions, ZFNs have been used to ablate HLA on CAR T-cells [22] and PD-1 in TILs [23]. Berdien et al. later delivered TALENs into T-cells with the goal of preventing the concurrent transgenic and endogenous TCR gene expression [24]. CRISPR has provided notable advantages over its predecessors. ZFN and TALEN rely on protein/DNA attachment for DNA binding and cleavage, resulting in the arduous process of engineering protein for each target sequence and limiting the number of DNA sites that can be effectively targeted [8]. This makes targeting a gene via CRISPR easier and more rapid since it only requires altering the gRNA sequence. It also has the added benefit of targeting multiple genes using several gRNAs. Finally, CRISPR is reportedly superior to ZFNs and TALENs in terms of efficacy and specificity, reducing the risk of off-target mutations and genotoxicity which are important considerations for clinical settings [9]. The emergence of CRISPR/Cas9 as a versatile technique has made precise, multiplex gene editing achievable with as little unwanted manipulation as possible. For instance, multiplex gene editing using CRISPR/Cas9 has allowed the production of TCRβ, PD-1, CTLA-4, and beta-2 microglobulin- (β2M)-deficient allogeneic CAR T-cells. These CAR T-cells exhibited no graft-versus-host disease (GVHD) and showed functionality both in vitro and in vivo [25].

## CRISPR/Cas9 to enhance CAR T-cells

On top of facilitating the development of therapeutic agents or being a therapy by itself, gene modification using the CRISPR/Cas9 technology has enabled scientists to genetically engineer animals to understand the etiology of various genetic disorders through disease modeling. Nevertheless, the therapeutic capabilities of CRISPR/Cas9 have also been established in many maladies. For instance, the *PSEN2*-mutated basal forebrain cholinergic neurons derived from induced pluripotent stem cells (iPSC) showed improved neural activity when the *PSEN2* mutation was corrected with CRISPR/Cas9 [26]; the mutant *PSEN2* gene is responsible for BFCN damage and is significantly correlated with early and late-onset familial Alzheimer’s disease. Another example is Duchenne muscular dystrophy (DMD) disease that is marked by DMD gene mutation at locus Xp21, causing a decrease in dystrophin protein production and subsequently, impaired muscular integrity. In vitro studies have utilized CRISPR/Cas9 for both gene knock-in in DMD patient-derived iPSCs [27] and large deletions in iPSC-derived cardiomyocytes [28] to successfully restore the synthesis of dystrophin. Also, in DMD-defective mdx mice, CRISPR/Cas9 has been shown to precipitate the correction of the DMD gene [29]. Hemophilia is another X-linked genetic disorder, which its treatment could benefit from genome editing. It is demonstrated that transfecting hemophilia B mice with AAV8 vectors carrying *S. aureus* Cas9 (SaCas9) and gRNA can successfully restore coagulation factor IX secretion of hepatocytes [30]. Lyu et al. further augmented the therapeutic potential of CRISPR/Cas9 in hemophilia by generating and transfecting a patient’s iPSCs with factor IX cDNA and subsequently, differentiating them into hepatocytes; the resultant cells expressed factor IX with no off-target mutations [31]. Transfection of wild-type (WT) iPSCs with specific gRNA to create homozygous cells with mutated CC chemokine receptor 5 (CCR5), called CCR5Δ32, holds promise for suppressing HIV-1 replication [32]. CCR5 is expressed on a variety of immune cells including macrophages, dendritic cells, and CD4+ T-cells, and is a key to HIV-1 cell invasion and infection. One study used CRISPR/Cas9 to remove the entire HIV-1 genome from infected CD4+ T-cells; this significantly reduced viral load and increased protection against HIV-1 [33].

The emergence of CRISPR/Cas9 sparked hope as an instrument with which current therapeutic approaches may be tweaked to their full potential. CAR T-cells, in particular, which have reinvigorated cancer immunotherapy in the past few years, can benefit from the versatility of the CRISPR/Cas9 platform. Despite the success of CAR T-cells in treating hematological malignancies, challenges such as cytokine release syndrome (CRS), T-cell exhaustion and durability, insertional oncogenesis, and the risk of GVHD remain. Also, the immunosuppressive and toxic tumor microenvironment and the inability of cells to penetrate the physical barrier of tumors greatly precludes the use of CAR T-cells in solid tumors [34]. By applying precise, targetable interventions, CRISPR/Cas9 could boost the therapeutic potential of CAR T-cells [35] (Table 2).

**Table 2:** CRISPR/Cas9-mediated gene therapy targets for enhancing TCR and CAR T-cell function

Cell type	Manipulation	Gene	Protein	Cancer Type	Summary	Advantage	Disadvantage
**CAR T-cell**	Universal CAR T-cells	TRAC/TRBC	TCRαβ	Acute Lymphoblastic Leukemia	Knocking out endogenous TCR chains reduces the likelihood of GVHD	Creates convenient, cheap, and rapid allogeneic CAR T-cells	HLA-I elimination could increase reactions from NK cells and lower the efficacy of therapy
		B2M		Acute Lymphoblastic Leukemia			
	Immune checkpoint blockade	PDCD1	PD-1	Hepatocellular Carcinoma	Singular or concurrent knockout of immune checkpoint inhibitors diminishes CAR T-cell exhaustion and increases cytotoxicity and proliferation	Creates more active and robust CAR T-cells	A potential risk of autoimmunity and off-target effects
		CTLA-4	CTLA-4	Acute Lymphoblastic Leukemia			
		LAG-3	LAG-3	Chronic Myelogenous Leukemia			
		Fas	CD94	Acute Lymphoblastic Leukemia			
		TGFBR2	TGF-β	Squamous Cell Carcinoma			
	Cytokine production	DGKA/DGKZ	DGK	Glioblastoma	Cytokines are pivotal in promoting CAR T-cell activation and function	Creates more persistent and cytotoxic CAR T-cell	A potential risk of CAR T-cell exhaustion/ limited knock-in efficacy
**TCR T-cell**	Universal TCR T-cells	TRAC/TRBC	TCRαβ	Acute Lymphoblastic Leukemia	Knocking out endogenous TCR chains reduces the likelihood of GVHD	Creates convenient, cheap, and rapid allogeneic TCR T-cells	HLA-I elimination could increase reactions from NK cells and lower the efficacy of therapy
		B2M		Acute Lymphoblastic Leukemia			
	Immune checkpoint blockade	PD-1	PD-1	Melanoma/ Renal Cell Carcinoma	Singular or concurrent knockout of immune checkpoint inhibitors diminishes TCR T-cell exhaustion and increases cytotoxicity and proliferation	Creates more active and robust TCR T-cells	A potential risk of autoimmunity and off-target effects
		CTLA-4	CTLA-4	Bladder Cancer			
		LAG-3	LAG-3	Chronic Myelogenous Leukemia			
		TGFBR2	TGF-β	Ovarian Cancer			
	Cytokine production	Dhx37	DHX37	Breast Cancer	Cytokines are pivotal in promoting CAR T-cell activation and function	Creates more active and robust TCR T-cells	A potential risk of TCR T-cell exhaustion/ limited knock-in efficacy
		Nr2f6	NR2F6	Colon Carcinoma			
		Gata3	GATA3	Colon Carcinoma			
		IFNG	IFN-γ	Melanoma			

### Universal CAR T-cells

Although autologous T-cells have shown promising results, in many cases, patients are not able to be treated or are treated with low-quality doses. This is especially the case with infant patients or patients whose lymphocyte repertoire is depleted due to myeloablative therapies or an underlying disease. Sometimes, poor results of CAR T-cell therapy can be attributed to an intrinsic defect of autologous T-cells [36]. In some diseases such as ALL or AML M3, the disease progression is so rapid that, because of time restriction, therapy with genetically engineered CAR T-cells becomes impossible. In other instances, the proliferative capability of patient T-cells in large-scale settings might be meager, leading to a low cell product; a clinically relevant dose in clinical settings requires billions of T-cells [37]. Lastly, in case of antigen mutation or antigen loss, the costly and lengthy process of CAR T-cell production must be repeated. The issue of autologous T-cells can be circumvented by developing universal genetically engineered CAR T-cells (UCART) derived from allogeneic healthy donors. Apart from a few occasions that allogeneic donor T-cells are used, such as bone marrow transplantation, the main source of T-cells is from the individual patient in an autologous setting. This is mainly due to the recognition of recipient alloantigens by donor TCRs, causing GVHD. These endogenous TCRs also mispair with α/β chains of genetically introduced TCR and compete for CD3 molecules [38]. Conversely, the recognition of donor HLA by the recipient’s immune cells results in graft rejection. Thus, TCR and HLA-I of universal allogeneic T-cells must be silenced. TCR silencing can be executed by knocking out the TCRα subunit constant (*TRAC*) or TCRβ gene (*TCRB*), eliminating the recognition of alloantigen of the recipient [39]. Additionally, CRISPR/Cas9 can be used to knock out β2M of donor CAR T-cells, a component that forms heterodimers with HLA-I and is required for HLA-I surface expression. The high capacity of multiplex CRISPR/Cas9 that simultaneously targets and knocks out TCR and β2M has created robust CD19-directed CAR T-cells with minimal risk of GVHD in mice with leukemia [25]. In 2017, by using multiplex CRISPR/Cas9, Liu et al. generated CAR T-cells with two (*TRAC* and *B2M*) or three (*TRAC*, *B2M,* and *PD-1*) disrupted genes and compared them with CD19 CAR T-cells. The triple knock-out (TKO) cells produced more IFN-γ and had higher cell lytic activity than double knock-out (DKO) and standard CAR T-cells. This can be explained by the knock-out of programmed cell death protein-1 (PD-1), which acts as a T-cell suppressor [39]. It is noteworthy that the lack of HLA-I (HLA-A, −B, −C, −E, −F, and -G) as a result of β2M elimination makes CRISPR/Cas9-modified CAR T-cells susceptible to in vivo natural killer (NK) cell-induced cytotoxicity, a phenomenon not addressed by the aforementioned studies. NK cells naturally recognize and kill cells lacking HLA-I via CD94/NKG2A and killer-cell immunoglobulin-like receptors (KIRs) [40]. Conventional strategies such as myeloablative conditioning, administration of NK-specific antibodies to block NK cells, matching donor-recipient HLA, or immunosuppression can be used to overcome this undesirable consequence; however, utilizing genetic engineering to simultaneously knock out HLA class I and force expression of non-classical HLA-I molecules (HLA-E, −F, −G) can prevent immediate NK cell cytotoxicity more efficiently [22, 41]. A CAR T-cell presenting β2M fused with HLA-E can evade recognition by NKG2A^+^ NK cells, but HLA-E is inherently unable to present endogenous antigens [42]. Moreover, since the KIR2DL1–4^+^ population of NK cells bind with HLA-C or HLA-G, knocking out these HLAs is unfavorable. Xu et al. generated CRISPR-edited pseudo-homozygous iPSCs from HLA heterozygous iPSCs. Using the same method, they also created HLA-C-retained iPSCs, in which HLA-A and HLA-B were disrupted. Both cells were able to evade T and NK cell recognition in vivo and in vitro [43].

### Immune checkpoint inhibitors

In normal, healthy conditions, after cellular immunity is activated and has successfully neutralized the invading pathogen, the immune system must be dampened to prevent T-cell over-activation and autoimmunity. Cancer cells evade the immune system by blocking immune checkpoint receptors (e.g. CTLA-4, PD-1, LAG-3, Tim-3, Fas, DGK, etc.) and abusing peripheral tolerance. In a process known as peripheral tolerance, checkpoint pathways are implemented to avert autoimmunity. Cytotoxic T-lymphocyte–associated antigen 4 (CTLA-4) and PD-1 are key immune checkpoints. CTLA-4 typically regulates T-cells in lymph nodes and is expressed on activated T-cells and regulatory T-cells (Treg). CTLA-4 binds to B7 ligands on antigen-presenting cells (APCs) with a higher affinity than CD28 (a receptor vital for secondary activation signal), leading to T-cell anergy [44]. PD-1, a hallmark of T-cell exhaustion, operates in the later stages of T-cell activation. It reduces T-cells’ activation and survival and also decreases secretion of IL-2, IFN-γ, and TNF-α upon binding to PD-L1/PD-L2 on macrophages and dendritic cells [45].

The expression of inhibitory ligands such as PD-L1 on tumor cells and their surrounding microenvironment creates an immunosuppressive milieu for the tumor [46]. In many cancers, the expression of PD-1 ligands is associated with poor prognosis [47]. Multiple studies have shown tumor regression in various cancers upon blocking the PD-1/PD-L1 axis [48, 49]. CTLA-4, LAG-3, and TIGIT receptors are also thought to synergize with PD-1 to counter T-cells, which is one of the major obstacle of CAR T-cells’ success with solid tumors [50]. This led to the approval of therapeutic monoclonal antibodies that target PD-1 (e.g., nivolumab) and CTLA-4 (e.g.*,* ipilimumab), and can be used in tandem with CAR T-cell therapy [51]. Unfortunately though, systemic blockade of immune checkpoint inhibitors might be associated with immune-related adverse effects throughout the entire body, involving the skin, liver, gastrointestinal tract, etc. [52]; CRISPR/Cas9 can be used to overcome this challenge by disrupting single or multiple immune checkpoint inhibitor genes of allogenic CAR T-cells instead of systemically administrating anti-immune checkpoint antibodies. In this aspect, functional apoptosis-resistant CAR T-cells have been created via simultaneous disruption of PD-1, CTLA-4, and Fas (CD94) [25]. In a study by Rupp et al., standard anti-CD19 CAR T-cells exhibited reduced degranulation and specific lysis upon co-culture with PD-1^+^/CD19^+^ K562 cells in comparison with CD19^+^ K562 cells. Infusing tumor-bearing NSG mice with standard anti-CD19 CAR T-cells showed a significantly higher mortality rate in the PD-1^+^/CD19^+^ group than the CD19^+^ group, further establishing the importance of PD-1 in CAR T-cell response [53]. Next, they disrupted the PD-1 gene (*PDCD1*) in CAR T-cells via Cas9 and reported increased degranulation and cytolytic capacity of these cells when cultured with PD-1^+^/CD19^+^ cells. In vivo, all the NSG mice were able to clear the tumor cells within 28 days.

Besides the inhibitory effects of ligand-receptor, CAR T-cells will have to contend with the intricate tumor microenvironment (TME) after infiltrating the solid tumor. TME is composed of various cancer-related cells and their inflammatory secretions, namely transforming growth factor-beta (TGF-β), which is abundantly produced by TME and cancerous cells. Among its pleiotropic functions, TGF-β in the TME suppresses T-cell function by inhibiting T-cell activation, expansion, and effector mechanisms [54]. Furthermore, in addition to downregulating CD8^+^ T-cell functional proteins (e.g. perforin, granzymes), it induces CD4^+^ T-cell differentiation toward Tregs, making it pivotal for tumor progression [55]. By knocking out TGF-β receptor II (*TGFBR2*) using CRISPR/Cas9, Tang et al. observed increased proliferation and functional markers of CAR T-cells in tumor xenograft-bearing mice. Interestingly, in *PDCD1-TGFBR2* DKO CAR T-cells, tumor eradication was more efficient in comparison to *TGFBR2*-edited CAR T-cells, emphasizing the importance of synergizing the knockout of multiple T-cell inhibitory pathways [56].

### CAR T-cells capable of cytokine secretion

**Fig. 2:**
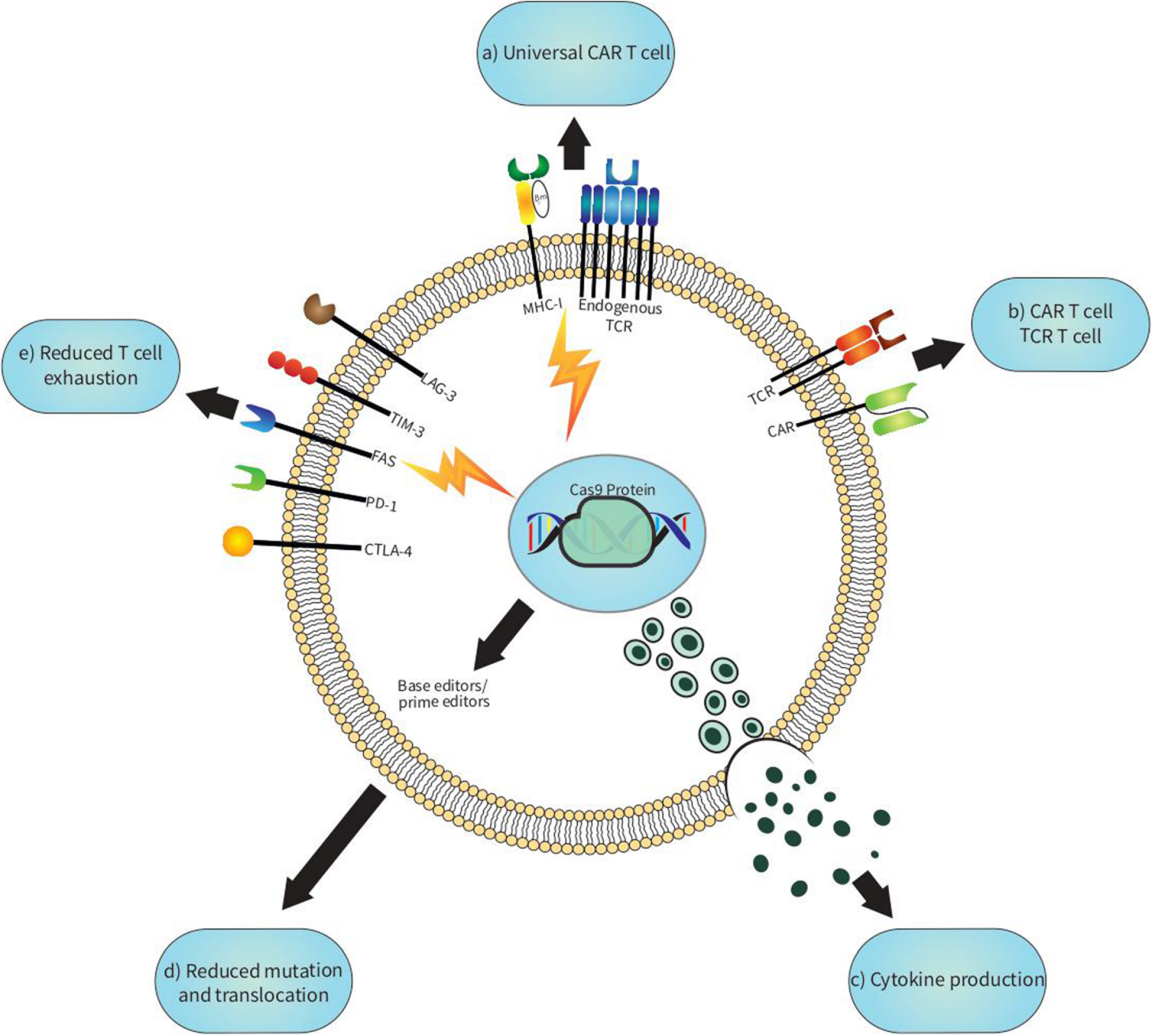
CRISPR/Cas9 is used to enhance the function of adoptive T-cell therapy. By knocking out endogenous TCR and MHC-I in off-the-shelf T-cells, the risk of graft-versus-host disease and host-versus-graft reaction is negated, respectively (**a**). CRISPR allows safe integration of TCR or CAR into T-cells for a uniform surface expression (**b**). Genes that propel cytokine production and genes that suppress them can be manipulated to increase cytokine production (**c**). By making use of base editors and prime editors, unwanted mutations can be denied to produce safer T cell products (**d**). Lastly, checkpoint inhibitors can be knocked out separately or in combination to create more durable and persistent T-cells (**e**)

For resting T-cells to activate, differentiate, and expand, a primary stimulation (TCR-CD3) and a secondary co-stimulation (CD28) are required. TCR signaling triggers downstream pathways, which among many other outcomes, leads to cytokine production (Fig. 2). Cytokines proceed to activate immune cells in return, as they are essential in modulating the function of T-cells. The strength of TCR stimulation vastly influences cytokine secretion [57]. IL-12 and IL-15 have been constitutively expressed by CAR T-cells to increase anti-tumor activity and long-term persistence, respectively [58, 59]. Also, IL-18 has been shown to promote IFN-γ production and CAR T-cell proliferation [60]. Hence, cytokine production can be artificially induced utilizing viral transduction; however, cytokine over-expression may lead to T-cell exhaustion or autoimmunity. CRISPR/Cas9 can alternatively be used to knock in the desired cytokine gene at a specific locus and bring its secretion under the strict control of an intrinsic promoter.

Diacylglycerol kinases (DGK) are enzymes that downregulate diacylglycerol (DAG), a second messenger formed downstream TCR activation, whose deletion enhances T-cell activity [61]. Jung et al. demonstrated how *DGKA/DGKB*-DKO anti-EGFRvIII CAR T-cells increased IFN-γ/IL-2 production and in vitro cytotoxicity and induced resistance to TGF-β and prostaglandin E2 [62]. These CAR T-cells also yielded promising results in terms of tumor regression in a glioblastoma xenograft mouse model. Silencing TGF-β via CRISPR/Cas9 dampens Forkhead box P3 (FOXP3), a master regulator responsible for Treg functioning, and subsequently inhibits the conversion of CAR T-cells into a Treg-related phenotype [56].

## Redirecting TCR T-cells using CRISPR/Cas9

TCR T-cells are another subset of ACT that have shown efficacy in the treatment of solid tumors, and same as CAR T-cells, TCR cells can be redirected to target a specific tumor-associated antigen (TAA) in a process called TCR gene therapy [63]. Although CAR T-cells have met success in hematological malignancies, they are not as advantageous in terms of recognizing a wide range of antigens. CAR T-cells can only be set to target a specific extracellular antigen, which constitutes < 1% of all proteins. Because TCR T-cells receive antigens from the entire proteome of cells via HLAs, they are able to recognize both the intracellular and surface antigens [64]. This form of antigen recognition is crucial since antigen loss on tumor cells is a cause for relapse after remission, and the expression of TAA antigens on normal cells could also hamper TCR T-cell application. Thus, discovering novel targets for TCR T-cells to extend the range of tumor specificity improves TCR gene therapy [65–67]. The second aspect to augment TCR gene therapy is developing optimal cell products with minimum side effects and high proliferative capacity [68]. Naturally, the promising results of CRISPR/Cas9 in improving CAR T-cell therapy have brought widespread attention to applying this technology for editing TCR-modified T-cells.

### Abrogation of endogenous T-cell receptors

TCRs are expressed as either αβ or γδ heterodimers, the former of which is the MHC-dependent conventional type and the latter can recognize malignant proteins with no MHC restrictions, making it a potent cancer immunotherapy candidate [69]. A major drawback of transgenic TCR T-cells is the expression of endogenous TCR. TCR forms a complex with CD3 to be expressed on the cell surface; thus, in the presence of an endogenous TCR, transgenic TCRs must compete to unify with CD3. There is also the possibility of endogenous and transgenic TCRs heterodimerization, creating four distinct TCRs and exhibiting lethal autoimmunity [70]. Also, considering the likelihood of TCRs provoking acute or chronic GVHD, the native TCR (*TRAC* or *TRBC*) of engineered T-cells must be silenced to achieve proper allogeneic T-cells. Gene editing techniques are among the chief methods to knock out the endogenous TCR expression [71]. Unlike TALEN, which is associated with subpar *TRAC* gene disruption rates and high T-cell toxicity, CRISPR/Cas9 is reportedly capable of producing highly pure TCR/CD3-negative cell populations [72]. CRISPR/Cas9 has been used to knock out endogenous TCRβ to create T-cells with increased expression of γδ and transgenic αβ TCRs, which stems from the low affinity of endogenous TCRα to dimerize with engineered TCRβ or γδ TCR. The resultant redirected T-cells showed a much greater polyfunctional response to a panel of cancer cell lines [73]. Although knocking out either *TRAC* or *TCRB* theoretically eliminates endogenous TCRαβ expression, TCR mispairs can occur following *TRAC* silencing. Morton et al. opted for the simultaneous *TRAC* and *TCRB* knockout in a single transfection using CRISPR/Cas9 RNPs, which resulted in efficient disruption in roughly 90% of the bulk T-cell population [74]. Instead of directly knocking out *TRAC*, Eyquem et al. inserted CD19 CAR in exon 1 of the *TRAC* locus, to not only silence *TRAC* but also to establish CAR expression, creating TCR negative CD19 CAR T-cells [75]. These CAR T-cells (*TRAC*-CAR) had boosted T-cell potency and uniform expression of CAR construct and had a significantly lower expression of PD-1, TIM3, and LAG3 exhaustion markers compared with conventional CAR T-cells. Alternatively, by using non-viral vectors that eliminate endogenous TCRαβ, TCRs that recognize New York esophageal squamous cell carcinoma 1 (NY-ESO-1) tumor-associated antigen can be inserted into *TRAC* locus instead of CAR and yield satisfactory effect [76].

### Crippling the inhibitory checkpoint molecules

By specifically disrupting the immune checkpoint genes, CRISPR/Cas9 has allowed TCR gene therapy to surmount its inhibitory effect while safeguarding the patient from the side effects of nonspecific immune checkpoint inhibitors. Several studies have attempted to disrupt PD-1 (the most studied inhibitory receptor) in primary T-cells of healthy and cancer-inflicted donors using CRISPR/Cas9. In 2015, Schumann et al. electroporated CD4^+^ T-cells with PD-1 Cas9 to target exon 1 of *PDCD1*, which created a knock-in and a frameshift mutation. This significantly reduced the surface expression of PD-1 [77]. In the same year, Su et al. disrupted PDCD1 in primary T-cells of both healthy and late-stage cancer donors, employing plasmid delivery [78]. Prolonged expansion of T-cells was unscathed, and the cells sustained PD-1 knockout over 21 days with enhanced cytotoxicity as well as IFN-γ production, a central cytokine in anti-tumoral responses [79]. Other studies took this further by disrupting PD-1 in antigen-specific cytotoxic T lymphocytes (CTLs) since PD-1 knock-out of primary T-cells could result in targeting auto-antigens in clinical settings. Su et al. were the first to describe PD-1 knockout of antigen-specific CTLs [78]. CRISPR/Cas9 was demonstrated to successfully disrupt the upregulation of PD-1 expression on EBV-LMP2A CTLs for Epstein-Barr virus (EBV)-positive gastric cancer and resulted in improved cytotoxicity. These cells experienced enhanced survival and restrained tumor growth in EBV-associated gastric cancer xenograft mouse models when combined with low-dose radiotherapy. In vitro, PD-1-abolished CTLs exhibited heightened production of IFN-γ, TNF-α, IL-2, and degranulation capacity, which are all hallmarks of CTL activation [78, 80]. Other studies on multiple occasions have substantiated the potential of CRISPR/Cas9-mediated disruption of *PDCD1* in boosting anti-tumor activities of CTLs [81] and even CD8^+^ memory CTLs [82]. As an indirect approach, PD-1 expression may be dampened in post-translational modifications by inhibition of *Fut8*, a fucosyltransferase gene responsible for PD-1 surface expression. Upon *Fut8* disruption in exhausted T-cells via CRISPR/Cas9, T-cells display stronger anti-tumor activity [83].

Taken together, these studies show the effect of CRISPR/Cas9-mediated disruption of PD-1 on vitalizing T-cell effector function; however, less research is devoted to disrupting other inhibitory receptors via CRISPR/Cas9. Indeed, blocking the *CTLA-4* gene in CD8^+^ CTLs with elevated PD-1/CTLA-4 expression enhances IFN-γ production and cytotoxicity against bladder cancer cells [84]. Zhang et al. demonstrated the feasibility of knocking out *LAG-3* in primary T-cells and CD19 CAR T-cells without any impairment in cell expansion [85]. However, in lymphoma xenograft models, *LAG-3*-KO CAR T-cells experienced no improvement in IL-2/IFN-γ production and cytotoxicity compared to control CAR T-cells, as well as no difference in tumor burden. This might be explained by the synergistic effect between LAG-3 and PD-1, and that both receptors must be blocked to reverse T-cell exhaustion [86].

### Boosting T-cell cytokine production

Each step of TCR stimulation and the subsequent downstream pathway can be tweaked to increase T-cell cytokine production. At the transcriptional level, transcription factors (TFs) such as nuclear factor kappa-light-chain-enhancer of activated B cells (NF-κB) are influential in IFN-γ production. Genome-scale screens to uncover immunotherapy targets have converged on DHX37, a helicase that modulates NF-κB and suppresses T-cell function [87]. CRISPR knockout of *Dhx37* has shown to upregulate granzymes C/D and IFN-γ and downregulate IL-6 and FOXP3 expression and increased CD8^+^ T-cell cytotoxicity [87]. Activator protein-1 (AP-1) is another TF pivotal in cytokine production, which is hamstrung by intracellular NR2F6. CRISPR-mediated *Nr2f6* ablation in combination with PD-L1 blockade in tumors resistant to anti-PD-L1 monotherapy has been able to increase IFN-γ secretion and delay tumor growth, suggesting a synergistic effect [88]. A viable strategy is knocking out TFs that inhibit cytokine production, such as GATA3, instead of promoting TFs that advance cytokine production. GATA3, which is highly expressed in CD8^+^Tim3^+^ TILs, is greatly involved in T-cell dysfunction and decreases IFN-γ and IL-2 production upon stimulation. In an in vivo study, CRISPR deletion of *GATA3* reversed this effect and *GATA3*-KO CD8^+^ T-cells decelerated tumor growth [89]. In addition to modifications of the transcription process, post-transcriptional regulations are also crucial for T-cells to regulate cytokine production. At the post-transcriptional level, cytokine production as proteins are partly controlled by RNA-binding proteins (RBPs) that recognize *cis*-elements in 5′ or 3′ of the untranslated region (UTR) of mRNA transcripts. Adenylate uridylate (AU)-rich elements (AREs) are *cis*-elements within 3′-UTR of IFN-γ, IL-2, and TNF-α mRNA. Cytokine mRNAs of resting T-cells are intrinsically unstable, and upon TCR and CD28 co-stimulation, RBPs bind to AREs at 3′-UTR, which leads to rapid mRNA stabilization [90]. Interestingly, elimination of 3′-UTR AREs from *IFNG* gene (ARE-Del) decoupled IFN- production from post-transcriptional modifications, precipitating IFN-γ production and delayed tumor growth in a melanoma mouse model [79]. These findings were corroborated by human MART-1 TCR T-cells, which displayed the same results likely due to 3′-UTR of *IFNG* being conserved between humans and mice [91].

It should be noted that the aforementioned proteins that impact cytokine production (e.g. DGK, DHX37, NR2F6, etc.) can alternatively be categorized as immune checkpoint inhibitors, and disrupting them along with other inhibitors, such as PD-1 and CTLA-4, may antagonize tumor resistance more efficiently. Nevertheless, precautions must be taken since the disruption of such genes may lead to hyperactive T-cells and autoimmunity. For example, although CRISPR/Cas9-mediated knock-out of *PTPN2* and *PTPN22* (negative regulators of TCR signaling) resulted in increased IFN-γ and IL-17 production, CD8^+^ T-cells exhibited autoimmunity in vivo [92].

## Current clinical applications of CRISPR/Cas9 in adoptive cell therapy

Over the past decade, many clinical trials have been carried out in order to actualize the potential of gene editing and translate this knowledge into clinical settings. Many clinical trials have been launched to assess the feasibility, safety, and efficacy of ZFNs, TALENs, and CRISPR gene editing platforms in multiple maladies such as β-thalassemia, AIDS, hemophilia B, mucopolysaccharidosis (MPS), sickle cell anemia, and a wide range of malignancies [93]. The first trial was initiated in 2009, which aimed to knock out *CCR5* from CD4^+^ T-cells for HIV using ZFNs (NCT00842634). In 2016 and 2017, the first TALEN (NCT02735083) and CRISPR (NCT02793856) human trials were registered, respectively. After a short period, the number of similar trials grew significantly, with a focus in favor of CRISPR/Cas9 application since this gene-editing platform was more simple, cost-effective and time-efficient compared with TALENs or ZFNs [94] (Table 3).

**Table 3:** Adoptive cell therapy (ACT) clinical trials using CRISPR/Cas9 technology for the treatment of cancer. Data extracted from https://clinicaltrials.gov/ (last accessed 6/3/2021)

Clinical Trial Number	Delivery Method	Phase	Target Gene/ Purpose	Cancer Type	Cell Type	Sponsor/Country	Date Posted/ Last Update	Recruitment Status
NCT02793856	N/A	I	*PDCD1*-KO	Metastatic Non-small Cell Lung Cancer	Primary T-cells	Peking University/China	6/8/2016 1/12/2021	Completed
NCT02863913	N/A	I	*PDCD1*-KO	Stage IV Invasive Bladder Cancer	Primary T-cells	Peking University/China	8/11/2016 3/6/2019	Withdrawn
NCT02867332	N/A	I	*PDCD1*-KO	Metastatic Renal Cell Carcinoma	Primary T-cells	Peking University/China	8/15/2016 3/6/2019	Withdrawn
NCT02867345	N/A	I	*PDCD1*-KO	Hormone Refractory Prostate Cancer	Primary T-cells	Peking University/China	8/15/2016 3/6/2019	Withdrawn
NCT03044743	N/A	I/II	PDCD1-KO	Multiple Neoplasms	Ant-EBV CTLs	The Affiliated Nanjing Drum Tower Hospital of Nanjing University Medical School/China	2/7/2017 5/2/2017	Recruiting
NCT03081715	N/A	I	PDCD1-KO	Esophageal Cancer	Primary T-cells	Hangzhou Cancer Center/China	3/16/2017 6/12/2019	Completed
NCT03538613	N/A	I/II	CISH-KO	Metastatic Gastrointestinal Cancers	TILs	National Cancer Institute/USA	6/11/2020 6/16/2020	Recruiting
NCT04089891	N/A	I/II	CISH-KO	Metastatic Gastrointestinal Epithelial Cancer	TILs	Masonic Cancer Center, University of Minnesota/USA	10/31/2019 8/3/2020	No Longer Active
NCT03398967	N/A	I/II	*TRAC, CD52*-KO	B cell Lymphoma/Leukemia	CD19/20- or CD19/22- UCART	Chinese PLA General Hospital/China	1/16/2018 1/16/2018	Recruiting
NCT03166878	Lentiviral (CAR) Electroporation (TCR/B2M)	I/II	*TRAC, TRBC, B2M*-KO	B cell Lymphoma/Leukemia	CD19-UCART	Chinese PLA General Hospital/China	5/25/2017 6/23/2017	Recruiting
NCT03690011	N/A	I	*CD7*-KO	T-cell Malignancies	CD7-CAR T-cells	Baylor College of Medicine/USA	10/1/2018 3/26/2021	Not yet recruiting
NCT03747965	N/A	I	*PDCD1*-KO	Multiple Mesothelin Positive Solid Tumors	Anti-mesothelin UCART	Chinese PLA General Hospital/China	11/20/2018	Unknown
NCT03545815	N/A	I	*TRAC, PDCD1-*KO	Multiple Mesothelin Positive Solid Tumors	Anti-mesothelin UCART	Chinese PLA General Hospital/China	6/4/2018 8/10/2020	Recruiting
NCT04035434	Electroporation	I	*TRAC*-KO	B cell Lymphoma/Leukemia	CD19-UCART (CTX110)	CRISPR Therapeutics AG/USA	7/29/2019 1/19/2021	Recruiting
NCT04037566	Lentiviral (CAR) Electroporation (HPK1)	I	*HPK1-*KO	B cell Lymphoma/Leukemia	CD19-CAR T-cells	Xijing Hospital/China	7/30/2019 7/30/2019	Recruiting
NCT03399448	Electroporation	I	*TRAC, TRBC, PDCD1*-KO	Multiple Malignancies	NYCE T cells	University of Pennsylvania/USA	1/16/2018 10/12/2020	Terminated

ACT for cancer has been one of the major focuses of recent gene-editing trials. The first CRISPR phase I clinical trial in humans was commenced in 2016 by investigators from the Chinese PLA General Hospital, which sought to inject *PD-1*-KO primary T-cells to stage IV metastatic non-small cell lung carcinoma patients (NCT02793856). Despite the use of primary non-specific T-cells, the study demonstrated low off-target effects and no severe treatment-related adverse effects [95]; however, the effectiveness of this study is obscure since the clinical efficacy of the treatment was not investigated [96]. In the same year, three other phase I clinical trials were started, evaluating *PD-1*-KO primary T-cells in Stage IV bladder cancer (NCT02863913), metastatic renal cell carcinoma (NCT02867332), and hormone-refractory prostate cancer (NCT02867345), all of which were later withdrawn. Even though TILs have shown efficacy in metastatic cancers, especially melanoma [97], and have demonstrated more specificity than primary modified T-cells, few trials are pursuing genetically edited TILs. Among them are two ongoing clinical trials that have opted to use CRISPR/Cas9 to disrupt *CISH* in TILs collected from gastrointestinal tumor sites (NCT04089891) (NCT04426669). Cytokine-induced SH2 (CISH) protein is a member of the suppressor of cytokine signaling (SOCS) family, which is induced in CD8^+^ T-cells upon TCR stimulation and inhibits T-cell anti-tumor function. In vivo models have already illustrated how *CISH* knockout leads to TIL expansion, function, and cytokine release, as well as tumor regression [98].

TILs and primary T-cells constitute only a small portion of CRISPR-mediated ACT trials, and the majority of clinical trials focus on TCR and especially, CAR T-cells. There are reportedly over 250 registered CAR T-cell clinical trials, of which a few have recently been dedicated to utilizing gene editing to augment CAR T-cell therapy [99]. Pre-clinical studies have shown the possibility of directing CD19-specific CAR into endogenous *TRAC* locus to decrease T-cell exhaustion and differentiation; these *TRAC*-CAR T-cells have reportedly outperformed conventional CAR T-cells [75]. Furthermore, there is an added benefit of fewer genetic manipulations associated with viral vectors, which translates to fewer translocations, insertional oncogenesis, etc. [100]. This 2-in-1 CAR-knock-in/TCR-knock-out is the aim of a trial that seeks to employ CD19 and CD20 or CD22 CARs integrated into endogenous *TRAC* locus (NCT03398967); these dual targeting UCARTs (CTA101) will likely solve the issue of antigen loss. In an open-label phase I study, Hu and colleagues attempted to evaluate the safety and efficacy of CRISPR/Cas9-engineered universal CD19/CD22 CAR-T cells in six patients with relapsed/refractory acute lymphoblastic leukemia. They reported no GVHD or gene editing-related adverse effects and no genotoxicity or chromosomal translocations upon CTA101 infusions; however, all patients developed CRS. This therapy also demonstrated prominent anti-tumoral activity with 83% of patients reaching complete remission after 28 days of CTA101 infusion [101]. Concerning T-cell malignancies, gene editing can be used to abrogate the expression of CD7. This attractive marker is highly expressive in T-cell tumors. Cooper et al. designed functional UCART7 to eliminate T-ALL in vitro*/*in vivo while avoiding the risk of GVHD [102]; this has become the bedrock of a current clinical trial in patients with T-cell leukemia or lymphoma (NCT03690011).

Immune checkpoint inhibitors are also being investigated in clinical settings. Trials are being carried out using CRISPR/Cas9 to disrupt PD-1 to produce mesothelin-directed CAR T-cells (NCT03747965) and UCARTs (NCT03545815). Lastly, a recent trial is investigating the efficacy of using edited endogenous hematopoietic progenitor kinase 1 (HPK1) in CD19-CAR T-cells; HPK1 is a kinase that negatively regulates TCR activation signals and is thus, a novel immunotherapy target [103].

As mentioned before, detecting the “right” antigen is imperative for a successful TCR T-cell therapy in order to increase anti-tumor efficacy and decrease treatment-related toxicity. For instance, trials using natural highly-reactive TCRs against a melanoma antigen, MART-1, have resulted in cancer regression but also severe toxicity [104]. To this end, a number of target antigens have been studied in pre-clinical and clinical studies, such as NY-ESO-1. This antigen, along with MAGE-A1 and MAGE-A3, is categorized as a cancer-testis (CT) antigen whose expression is limited to male germ cells and cancer cells (e.g. myeloma, sarcoma, and melanoma) [105]. As of 2021, the only first-in-human clinical trial to assess the feasibility, safety, and clinical activity of TCR gene therapy was NCT03399448, which aimed to use NY-ESO-1/LAGE-1-specific transgenic TCRs. Taking advantage of CRISPR, multiplex gene editing was performed to silence endogenous TCRα/β (45% editing frequency for *TRAC* and 15% for *TRBC*) and knockout PD-1 (20% editing frequency for *PDCD1*) to create triple-edited T-cells and increase safety and efficacy, respectively. Using lentiviral transduction, transgenic TCRs, which were detectable in 2–7% of the final product cells, were introduced into T-cells (named NYCE T-cells). Three patients were infused with functional NYCE T-cells with no serious adverse effects and negligent humoral immunity versus Cas9, which can be attributed to diminishing Cas9 protein levels in the pre-infusion expansion of T-cells [106]. The NYCE cells’ impressive trafficking to bone marrow (BM) and tumor sites was comparable to their blood levels, which persisted for up to 300 days in the blood; this was considered a step up compared to previous trials [107, 108]. Safe off-target mutations were detected, and inevitable chromosomal translocations were reported in NYCE T-cells; however, the frequency of translocations dwindled throughout large-scale expansion and post-infusion, indicating that rearrangements in engineered T-cells do not demonstrate proliferation advantage over a long expansion period in vivo [106]. Although the patients did not experience abatement in cancer, the study further demonstrated the safety of multiplexed CRISPR/Cas9. Additional studies with more subjects, higher gene-editing efficiencies, and more current reagents and protocols are needed to further elucidate the efficacy of CRISPR/Cas9 [109].

## Challenges and limitations of CRISPR/Cas9

The advent of CRISPR/Cas9 is raising the expectation of eliminating cancer on an unprecedented scale. Still, some lingering issues from both the cancer and gene therapy aspect need to be addressed. For instance, the heterogeneity and perpetual evolution of cancer cells, and their transitional mutation profile make treatment difficult for any gene therapy approach. These issues relate to either ACT therapy in general or CRISPR gene editing. Some of the challenges regarding CRISPR/Cas9 efficacy are as follows:

1. ***Off-target toxicity:*** Apart from the GVHD side effect, ACT-related toxicities, especially in the case of CAR-T cells, is a major concern. TCR and CAR T-cell therapies respond only to their predestined antigens with no attention to the antigen-carrier cell, sometimes resulting in on-target off-tumor side effect. In anti-CD19 CAR T-cell therapy, for instance, B-cell aplasia is frequently observed since normal B-cells also express CD19. CRISPR can tackle on-target off-tumor side effect issue by ablating target antigens on normal cells. CD5 and CD7 are TAAs expressed on not only T-cell tumors but also on the surface of normal T-cells. Thus, targeting CD7 using anti-CD7 CAR T-cells results in the self-killing of CAR T-cells (fratricide) and suboptimal therapy. One simple approach would be to knock out CD7 [110] and CD5 [111] to create fratricide-resistant T-cells. Furthermore, traditional CAR T-cell therapy of acute myeloid leukemia (AML) can target CD33 or CD7, which are expressed on normal hematopoietic stem cells (HSCs) and tumor cells [112]. This ultimately results in myeloablation of the BM and mortality in leukemia patients. Some studies have attempted to bypass this problem by eliminating CD33 on donor HSCs using CRISPR/Cas9 combined with anti-CD33 CAR T-cells [113, 114]. This culminates to CAR T-cells eradicating CD33-bearing tumor cells, while CD33-null HSCs proceed to repopulate the BM. Creative approaches such as these provide scientists with new insight to confront cancer.
2. ***Cas9-related immunogenicity:*** The CRISPR/Cas9 system as a whole has been shown to trigger both innate and adaptive immunity. The CRISPR crRNA in Cpf1 (Cas12a) and the triphosphate group at the 5′ end of gRNA in Cas9 can engender RNA-sensing type I IFN-mediated innate immune response, resulting in T-cell death and low therapeutic efficiency [115]. The removal of the triphosphate group creates a 5′-OH group that reverses this cytotoxic effect while still being highly efficient to knock out *CCR5* in primary human CD4^+^ T-cells [116]. However, further studies are required to minimize innate immune response to gRNA, mRNA, and delivered DNA. Since the most common Cas9 orthologs used in CRISPR are derived from *S. aureus* (SaCas9) and SpCas9, which are abundantly colonized in human populations, a pre-existing adaptive immunity against Cas9 is formed [117]. In single-dose therapies, the accumulation of antibodies may not pose a threat but in circumstances of repeated therapies or developed immunity, monitoring of clinical conditions is necessary. Still, gradual Cas9 protein degradation and surface presentation on MHC elicits CTL-mediated cellular immunity, eventually rendering gene therapy ineffective [118]. The delivery method is another culprit in this scenario, as adenoviral vectors are extremely immunogenic and induce hepatotoxicity and inflammation. Current approaches to counter immunogenicity include deimmunization by mutating antigenic peptides to humanized neoantigens (epitope masking), decreasing immunological exposure to Cas9 by governing its expression or inducing degradation, or ultimately, immunosuppression [119].
3. ***Off-target mutations:*** Ideally, a gene-editing system must perform its function with high efficacy and no bystander mutations; however, this is rarely the case with not only CRISPR but with any other editing platforms. Mutations have been reported in multiplex gene editing of CAR T-cells [39]. Using whole-genome sequencing, one study claimed that gene editing resulted in hundreds of unexpected mutations in CRISPR/Cas9-treated mice [120]. This is of special importance in multiplex CRISPR genome editing in which chromosomal translocation among DSB sites is more likely to occur. The off-target effects of gene therapy, however, do not prevent their application in the clinic, especially if they are low in frequency and on a non-coding genome (NCT03399448). Off-target mutations are chiefly due to PAM or gRNA seed sequence mismatches. The specificity of the Cas9-gRNA complex is influenced by the nucleotides of the seed sequence. For instance, U-rich seed sequences, and very high or very low seed GC content increase and decrease gRNA specificity, respectively. The sequence of PAM is also important. Canonically, NGG (N being A, T, G, or C) is the predominant PAM sequence, but NRG (R being G or A) is also considered for the type II CRISPR system [100]. Assays and software have assisted with predicting off-target DNA breaks in combination with DNA sequencing in vitro and in cell models [121]. Novel systems have been introduced to monitor DSBs and Cas activity in vivo [122, 123]. Although these methods are highly sensitive in detecting small mutations and indels, the threat of chromosomal rearrangements, such as deletions, inversions, and translocations still exists. Giannoukos et al. developed a method named UDiTaS to overcome the limitations of PCR and next-generation sequencing by quantitatively detecting both indels and large translocations [124]. To accomplish this, a customized Tn5 transposon comprised of an i5 forward primer, a sample barcode, and a unique molecule identifier (UMI) was tagmented into the edition spot. Then, a first PCR round was conducted using the i5 primer and a second PCR with an additional sample barcode and i7 reverse primer to complete the Illumina sequencing library. The resultant UMI barcode was used to detect editing, inversions, and large deletions [124].
  Base editors (BEs) and prime editors (PE) are single-base-pair editing systems that limit the off-target mutations of CRISPR to increase the safety of the technology by eliminating the need for donor DNA templates and DSBs altogether. BEs, comprised of a Cas enzyme and a DNA-modifying deaminase, induce synthetic mutations in a small length of 5 bp. Cytosine BEs and adenine BEs surgically modify a desired gene to change gene function or disrupt its expression by installing four transition mutations (C → T, T → C, G → A, A → G) [125]. The precise, single nucleotide alteration means that the changes are predictable and easy to trace. Also, in the absence of DSBs, the risk of chromosomal rearrangements and translocations are miniscule. First introduced by Anzalone et al., PEs rely on a Cas9 variant fused to an engineered reverse transcriptase and a prime-editing guide RNA (pegRNA) within the construct [126]. There is a guide sequence on the 5′ end of pegRNA and a DNA-binding primer and an RNA template to synthesize the edited sequence. PEs are able to perform the four transition mutations, the eight transversion mutations (G → T, A → C, A → T, G → C, C → G, C → A, T → A, A → T), and insertions and deletions. This platform is more versatile than BEs and more efficient than HDR templates [127]. Webber et al. successfully performed *TRAC*, *B2M*, and *PDCD1* abrogation in primary human T-cells, and the resultant CD19 CAR T-cells had significantly reduced DSB and translocation frequencies compared to Cas9 [128]. Similarly, other studies have used BEs to knockout multiple alloreactivity-associated genes (*PDCD1, CIITA, TRAC, CD7, B2M, CBLB*) [129] or to remove TCR/CD3/CD7 to create anti-CD3/CD7 fratricide-resistant CAR T-cells [130]. Conclusively, diminishing off-target mutations of CRISPR editing must be considered for future developments, and off-target detection assays are necessary to ensure safety for clinical trials.
4. ***A risk of malignancy:*** Apart from the undesired consequences of CRISP/Cas9 on DNA, its effect on protein expression has also been studied. P53 is a pivotal tumor suppressor whose gene mutation not only results in faulty tumor suppression but also provides oncogenic functions to cancerous cells [131]. It was discovered that when Cas9-mediated DNA breakage is detected in human retinal epithelial cells, p53 signals DNA damage response and arrests the cell cycle [132]. CRISPR/Cas9 editing is more competent when disrupting *TP53* and, at the same time, monitoring its function. Through a transiently suspending DNA repair system, the rate of HDR recombination from donor templates is accelerated. Nevertheless, this may lead to the escape of cells with damaged DNA in the editing process, unwanted DNA rearrangements and proto-oncogene mutations, subsequently leading to increased risk of cancer.
5. ***Ethical concerns:*** Not all the drawbacks of gene editing are centered on its biological aspects and some limitations are more general. Ever since a team of Chinese scientists reported the possibility of gene editing in a human embryo, a great deal of controversy was sparked around “designer babies.” Genetic manipulation can be performed on germline (e.g. human eggs and sperm or embryo) and somatic cells alike. Genetic changes in germline cells have the benefit of being perpetually inherited, which is exciting in the case of treating diseases but it can also engender inequalities and discrimination. Moreover, the risks associated with the exceeding use of genetic manipulation could involve all life forms, causing ecological imbalance and animal welfare issues. Bioethical dilemmas are bound to occur in the instance of both failed and successful applications of gene editing; however, their discussion is out of the scope of this article [123–125].

## Conclusions

To conclude, CRISPR/Cas9 is set to revolutionize the world with the treatment and elimination of illnesses being only one of its prospects. All facets of medical, basic, industrial, and agricultural sciences will benefit from these molecular scissors that won the 2020 Chemistry Nobel Prize. Thanks to CRISPR, gene therapy is rapidly growing and a vast array of new methods, such as Cas-based RNA targeting, are being discovered to create hyper-accurate and next-generation CRISPR systems [133]. Though ACT has been a novel and sensational approach in battling cancer, it has faced some hurdles ever since its introduction; being limited to hematological malignancies, T-cell exhaustion and loss of function, allogeneic T-cell-associated GVHD, and manufacturing costs are to name a few. The versatile CRISPR platform, although not entirely infallible, has enabled ACT to surmount these barriers in pre-clinical studies. By taking advantage of CRISPR technology to eliminate MHC and endogenous TCR expression, potential GVHD associated with allogeneic T-cell therapy is minimized. CRISPR may also be used to optimize T-cell function and reduce exhaustion by disrupting immune checkpoint proteins. More potent therapeutic T-cells translates to lower doses of cells, less toxicity, and less cost. With the aid of CRISPR, large-scale use of universal CAR T-cells as an off-the-shelf therapeutic option, especially for patients with little time, is more possible; these cells are expected to have their manufacturing costs significantly reduced. Advanced methods of precision editing will help overcome the off-target mutations and toxicities associated with ACT. Trials are still in their early phase but with more and more clinical trials being green-lit and the preliminary results of several trials demonstrating feasibility and efficacy, the future of cancer treatment seems bright.

## Data Availability

Not applicable.

## Abbreviations

AAV: Adeno-associated virus
ACT: Adoptive T-cell therapy
CAR: Chimeric antigen receptor
CRS: Cytokine release syndrome
crRNA: CRISPR pre-RNA
CTLs: Cytotoxic T lymphocytes
CRISPR: Clustered regularly interspaced short palindromic repeats
DDR: DNA damage response
DSB: Double-stranded break
DNA: Deoxyribonucleic acid
GVHD: Graft-versus-host disease
gRNA: Single-guide RNA
HDR: Homology-directed repair
HLA: Human leukocyte antigen
MHC: Major histocompatibility complex
NHEJ: Non-homologous end joining
RNA: Ribonucleic acid
RVDs: Repeat variable di-residues
TALENs: Transcription activator-like effector nucleases
TCR: T-cell receptor
TILs: Tumor-infiltrating lymphocytes
ZFNs: Zinc finger nucleases
